# Concurrent cisplatin and 5-fluorouracil *versus* concurrent cisplatin and docetaxel with radiotherapy for esophageal squamous cell carcinoma: a propensity score-matched analysis

**DOI:** 10.18632/oncotarget.9301

**Published:** 2016-05-11

**Authors:** Peng Zhang, Mian Xi, Qiao-Qiao Li, Yong-Hong Hu, Xiaobo Guo, Lei Zhao, Hui Liu, Shi-Liang Liu, Li-Ling Luo, Qing Liu, Meng-Zhong Liu

**Affiliations:** ^1^ Department of Radiation Oncology, Sun Yat-sen University Cancer Center, Guangzhou, People's Republic of China; ^2^ Collaborative Innovation Center for Cancer Medicine, Guangzhou, Guangdong, People's Republic of China; ^3^ Guangdong Esophageal Cancer Research Institute, Guangzhou, Guangdong, People's Republic of China; ^4^ Department of Statistical Science, School of Mathematics & Computational Science, Sun Yat-Sen University, Guangzhou, People's Republic of China; ^5^ Department of Ophthalmology, University of Melbourne, Melbourne, Victoria, Australia; ^6^ Department of Medical Statistics and Epidemiology, School of Public Health, Sun Yat-sen University, Guangzhou, Guangdong Province, People's Republic of China

**Keywords:** esophageal cancer, chemoradiotherapy, propensity score, prognostic factor

## Abstract

The optimal concurrent chemotherapy regimen with radiotherapy for esophageal cancer is unknown. Here, we compared the survival outcomes and toxicity of definitive chemoradiotherapy with either cisplatin/5-fluorouracil (PF) or docetaxel/cisplatin (DP) in patients with unresectable esophageal squamous cell carcinoma (ESCC). In this study, we identified 317 patients with ESCC who received PF or DP concurrently with definitive radiotherapy. PF group patients received two cycles of cisplatin (60 mg/m^2^) and 5-fluorouracil (300 mg/m^2^) at 4-week intervals during radiotherapy. DP group patients received a concurrent three-weekly schedule of docetaxel (60 mg/m^2^) and cisplatin (80 mg/m^2^) or cisplatin (25 mg/m^2^) and docetaxel (25 mg/m^2^) weekly. The overall survival (OS) and progression-free survival (PFS) were compared using propensity score (−adjusted, −weighted, −stratified, and −matched) analyses. A sensitivity analysis was performed to examine the impact of unmeasured confounders. Inverse probability of treatment weighting for propensity score demonstrated an improvement in OS and PFS with DP group in comparison with PF group (hazard ratio, 0.700; 95% CI, 0.577-0.851) and similar results were achieved with propensity score matching and stratification. Grade 3-4 esophagitis was more common (16/102 *vs*. 4/102) and grade 3-4 thrombopenia and skin toxicity were less common (3/102 *vs*. 10/102; 7/102 *vs*. 19/102; respectively) in the PF group than the DP group. In conclusion, concurrent chemoradiotherapy with the DP regimen resulted in better OS and PFS compared to concurrent PF regimen with tolerable toxicities in ESCC patients. Prospective randomized trials are required to confirm the efficacy of the DP regimen.

## INTRODUCTION

Esophageal cancer represents a considerable health problem globally and has a 5-year survival rate of approximately 17% [1]. Chemoradiotherapy is the standard treatment for unresectable esophageal cancer. Randomized controlled trials have established the superiority of concurrent chemoradiotherapy over radiotherapy alone in localized esophageal cancer [2, 3]. The most commonly-applied regimen for radiosensitization in esophageal cancer is the doublet combination of cisplatin and 5-fluorouracil (PF). However, the outcomes of this regimen remain unsatisfactory in terms of local control, toxicity and overall survival benefit [4, 5, 6]. Therefore, more effective regimens need to be investigated to improve the prognosis of patients with locally advanced esophageal cancer.

Several studies have reported encouraging results for docetaxel and cisplatin (DP); this regimen has been widely used in esophageal cancer and applied concurrently with radiotherapy in recent years [7–9]. However, no large randomized phase III clinical trial has yet directly compared the concurrent PF and DP regimens combined with radiotherapy in esophageal cancer. To gain insight into the relative efficacy and toxicities of the PF and DF regimens, we retrospectively reviewed patients diagnosed with esophageal squamous cell carcinoma (ESCC) treated at our institution with definitive radiotherapy combined with either concurrent PF or DP.

## RESULTS

Of the 317 patients, 254 were male (80.1%) and 63 were female (19.9%). The demographic and clinical characteristics of the cohort are listed in Table 1. According to the 6th edition of the TNM staging system, 22 patients were classified as stage II (6.9%), 165 as stage III (52.1%), and 130 as stage IVa (41.0%).

**Table 1:** Characteristics of patients treated with PF versus DP in the observational data set and in patients after propensity score matching

Characteristic	Observational dataset (*n* = 317)			Propensity score–matched dataset (*n* = 204)		
	PF	DP	Standardized	PF	DP	Standardized
	(*n* = 156), %	(*n* = 161), %	Difference	(*n* = 102), %	(*n* = 102), %	Difference
**Age, years**			−0.19			0.02
**Mean ± SD**	56.4± 8.4	58.0± 7.8		57.5 ± 7.8	57.3 ± 8.0	
**Sex**			0.09			0.05
** Male**	128 (82.0)	126 (78.3)		82 (80.4)	80 (78.4)	
**Female**	28 (18.0)	35 (21.7)		20 (19.6)	22 (21.6)	
**Time of diagnosis**						
**2002-2005**	82 (52.6)	26 (16.2)	0.83	30 (29.4)	26 (25.5)	0.08
**2006-2009**	39 (25.0)	63 (39.1)	0.30	37 (36.3)	37 (36.3)	0
**2010-2013**	35 (22.4)	72 (44.7)	0.49	35 (34.3)	39 (38.2)	0.08
**Weight loss, %**			0.01			0.05
**Mean ± SD**	3.7 ± 5.6	3.8 ± 5.4		4.1 ± 5.9	3.8 ± 5.2	
**ECOG PS**			0.04			0.05
**0**	135 (86.5)	137 (85.1)		83 (81.4)	85 (83.3)	
**1–2**	21 (13.5)	24 (14.9)		19 (18.6)	17 (16.7)	
**Charlson score**						
**≥1**	55 (35.3)	42 (25.5)	0.21	35 (34.3)	31 (30.4)	0.08
**<1**	101 (64.7)	119 (73.9)		67 (65.7)	71 (69.6)	
**Platelets, ×109/L**			0.21			0.01
**Mean ± SD**	262.1 ± 73.3	245.1 ± 90.4		252.2 ± 65.7	251.4 ± 99.2	
**Serum albumin, g/L**			0.11			0.10
**Mean ± SD**	42.3 ± 3.7	41.9 ± 3.9		42.4 ± 3.6	42.0 ± 4.0	
**Hemoglobin, g/L**			0.14			0.09
**Mean ± SD**	139.5 ± 26.7	136.5 ± 16.4		137.4 ± 16.5	135.1 ± 16.5	
**Histologic grade**						
** Well differentiated**	37 (23.7)	43 (26.7)	0.06	29 (28.4)	32 (31.4)	0.06
** Moderately differentiated**	91 (58.3)	72 (44.7)	0.27	53 (52.0)	51 (50.0)	0.04
** Poorly/undifferentiated**	28 (18.0)	46 (28.6)	0.25	20 (19.6)	19 (18.6)	0.02
**Tumor location**						
**Upper third**	71 (45.5)	74 (46.0)	0.01	48 (47.1)	48 (47.1)	0
**Middle third**	77 (49.4)	72 (44.7)	0.09	49 (48.0)	44 (43.1)	0.09
**Lower third**	8 (5.1)	15 (9.3)	0.16	5 (4.9)	10 (9.8)	0.08
**TNM stage**						
**II**	7 (4.5)	15 (9.3)	0.19	5 (4.9)	7 (6.9)	0.08
**III**	87 (55.8)	78 (48.5)	0.15	55 (53.9)	49 (48.0)	0.10
**IVa**	62 (39.7)	68 (42.2)	0.05	42 (41.2)	46 (45.1)	0.08

Abbreviations: DP: docetaxel and cisplatin; PF: cisplatin and fluorouracil; SD, standard deviation; ECOG, Eastern Cooperative Oncology Group; PS, performance status.

### Receipt of PF *versus* DP

Of the 317 patients in this cohort, the PF regimen was used concurrently in 49.2% (*n* = 156) patients and the DP regimen in 50.8% (*n* = 161). Analysis of several clinicopathological factors, including age, sex, performance status, Charlson score, tumor location, histologic grade, N classification, M classification and TNM stage, did not reveal a significant association between any factor and the receipt of PF or DP (Table 2). In multiple logistic regression analysis, treatment at an earlier era and a lower histologic grade were associated with the receipt of PF (Table 2).

**Table 2:** Factors associated with receipt of PF versus DP

Factors	No.	Univariate analysis			Multivariate analysis		
		OR	95 % CI	*P*	OR	95 % CI	*P*
**Age (yr)**							
** <58**	157	Ref	−				
** ≥58**	160	1.271	0.817 - 1.975	0.287			
**Sex**							
**Male**	254	Ref	−				
**Female**	63	0.788	0.452 - 1.371	0.398			
**Era of diagnosis**							
**2002-2005**	108	Ref	−		Ref	−	
**2006-2009**	102	5.095	2.810 - 9.237	< 0.001	5.135	2.755 - 9.571	< 0.001
**2010-2013**	107	6.488	3.567 - 11.799	< 0.001	6.413	3.372 - 12.198	< 0.001
**ECOG PS**							
**0**	272	Ref	−				
**1–2**	45	1.126	0.599 - 2.119	0.713			
**Charlson score**							
**<1**	220	Ref	−		Ref	−	
**≥1**	97	0.648	0.401 - 1.049	0.077	0.992	0.578 - 1.703	0.976
**Tumor location**							
**Upper third**	145	Ref	−				
**Middle third**	149	0.897	0.568 - 1.418	0.642			
**Lower third**	23	1.799	0.719 - 4.504	0.210			
**Histologic grade**							
** Well differentiated**	80	Ref	−		Ref	−	
** Moderately differentiated**	163	0.681	0.398 - 1.165	0.161	0.509	0.276 - 0.936	0.030
** Poorly/undifferentiated**	74	1.414	0.743 - 2.690	0.292	0.911	0.439 - 1.891	0.803
**T stage**							
** T2**	44	Ref	−		Ref	−	
** T3**	158	0.452	0.220 - 0.929	0.031	0.582	0.248 - 1.368	0.215
** T4**	115	0.300	0.142 - 0.634	0.002	0.528	0.210 - 1.326	0.174
**N stage**							
** N0**	45	Ref	−				
** N1**	272	1.345	0.714 - 2.537	0.359			
**M stage**							
** M0**	187	Ref	−				
** M1**	130	1.109	0.708 - 1.735	0.652			
**TNM stage**							
**II**	22	Ref	−		Ref	−	
**III**	165	0.418	0.162 - 1.079	0.072	0.718	0.219 - 2.354	0.584
**IVa**	130	0.512	0.196 - 1.338	0.172	0.709	0.216 - 2.325	0.570

### Survival outcomes and propensity score-matched analysis

The median follow-up periods for the patients in the PF and DP groups were 24 (range, 4-128 months) and 21 months (range, 3-99 months), respectively. The 1, 2, and 3-year OS rates for the entire cohort were 77.4%, 48.9%, and 32.8% (median OS: 18.0 months). The 1, 2, and 3-year PFS rates for the whole group were 67.6%, 40.4%, and 29.5% (median PFS: 15.0 months). In multivariate cox regression analysis of the whole cohort, the treatment group was a significantly prognostic factor of OS but not an independent prognostic factor associated with PFS (Table 3).

**Table 3:** Effect of concurrent chemotherapy regimens on OS and PFS in esophageal cancer patients receiving chemoradiotherapy

Model	Sample size	OS			PFS		
		HR	95% CI	*P* value	HR	95% CI	*P* value
Unadjusted	161 vs. 156	0.697	0.528-0.920	0.011	0.784	0.599-1.027	0.077
Propensity score–based models							
Stratified	161 vs. 156	0.677	0.493-0.928	0.016	0.751	0.553-0.945	0.036
Weighted (IPTW)	161 vs. 156	0.700	0.577-0.851	0.003	0.759	0.628-0.918	0.014
Matched^a^	102 vs.102	0.641	0.464-0.887	0.007	0.663	0.474-0.928	0.016

Abbreviations: OS, overall survival; PFS, progression-free survival; HR, hazard ratio; CI, confidence interval; IPTW, inverse probability of treatment weighting.

a Adjusted hazard was derived from the Cox proportional hazards marginal structural model.

**Figure 1:**
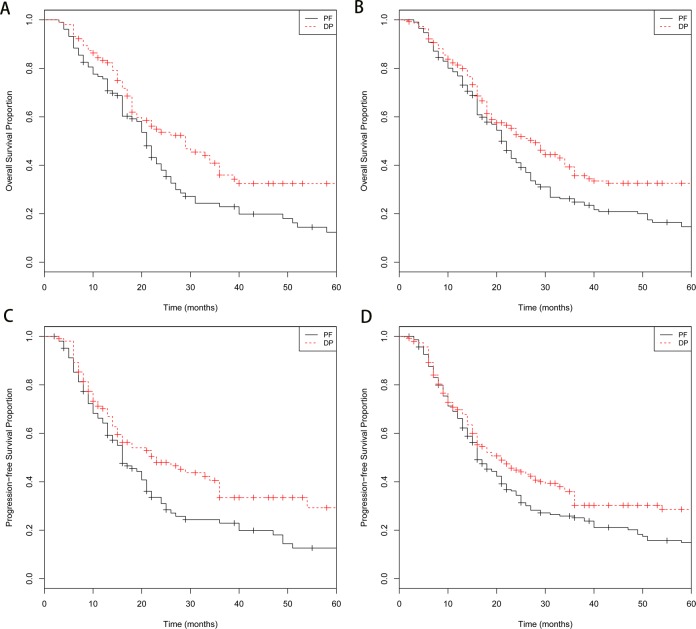
Kaplan-Meier survival curves for patients stratified by treatment with docetaxel/cisplatin (DP) *versus* fluorouracil/cisplatin (PF) **A.** Kaplan-Meier survival curves of overall survival (OS) time in the propensity score-matched cohort (*P* = 0.009); **B.** inverse probability of treatment weighted (IPTW)-adjusted Kaplan-Meier estimates of OS time curves in the whole cohort (*P* < 0001); **C.** Kaplan-Meier survival curves of progression-free survival (PFS) time in the propensity score-matched cohort (*P* = 0.010); **D.** IPTW-adjusted Kaplan-Meier estimates of PFS time curves in the whole cohort (*P* = 0.004).

Adjusting or matching by propensity score achieved adequate balance between DP and PF group (Table 1). Compared with PF group, the DP group had significantly better OS (log-rank, *P* = 0.009; Figure 1A) and PFS (log-rank, *P* = 0.010; Figure 1C) in propensity score matched cohort. IPTW-adjusted Kaplan-Meier estimates of OS and PFS time probabilities in the whole cohort were also computed for DP and PF groups and revealed the similar results (OS, IPTW log-rank, *P* < 0001; Figure 1B; PFS, IPTW log-rank, *P* = 0.004; Figure 1D). Analyses stratified by propensity score quintile revealed that the DP group was associated with improved OS (HR, 0.677; 95% CI, 0.493-0.928; *P* = 0.016) and PFS (HR, 0.751; 95% CI, 0.553-0.945; *P* = 0.036 ). The marginal structural models analyses yielded similar results (Table 3). The DP group also had a significantly better PFS in comparison with PF group (Table 3). In the propensity score matched cohort, the marginal structural models also revealed that the TNM stage (HR, 1.619; 95% CI, 1.274 to 2.246; *P* < 0.001) and tumor location (HR, 1.428; 95% CI, 1.090 to 1.871; *P* = 0.009) were independent factors associated with OS. Besides the treatment group, the significantly prognostic factor influencing PFS also included TNM stage (HR, 1.667; 95% CI, 1.273to 2.185; *P* < 0.001).

As is shown in Table 4, the results was relatively robust to the effects of unmeasured confounders in the sensitivity analysis. Baseline HR for this analysis was from the marginal structural model. We fixed the prevalence of binary confounder in DP group to be 0.2, and varied the prevalence of unmeasured confounders in PF group and the strength of unmeasured confounder effects (strong confounder: HR = 1.5; moderate confounder: HR = 1.3) to explore the extent of imbalance on the unmeasured confounder between two groups that could affect the statistical significance of results. For example, assuming an HR of 1.5 (strong confounder), unmeasured confounders would not eliminate the significant OS advantage of DP group even under the circumstances of 40% difference in the prevalence of unmeasured confounder (60% *vs.* 20%). The sensitivity analysis indicated that the models are robust and that the unmeasured confounder would have to be markedly associated with survival and differential prevalence between two groups to alter the significance of our results.

**Table 4:** Sensitivity analysis of the effects of unmeasured confounder on HRs of OS and PFS

Prevalence of UC		UC HR	OS		PFS	
DP group	PF group		HR	95% CI	HR	95% CI
0.4	0.2	1.3	0.678	0.490-0.938	0.701	0.501-0980
0.4	0.2	1.5	0.700	0.506-0.868	0.724	0.518-0.988
0.6	0.2	1.3	0.714	0.517-0.988	0.739*	0.528-1.033
0.6	0.2	1.5	0.758	0.548-0.949	0.784*	0.561-1.096
0.8	0.2	1.3	0.751*	0.543-1.038	0.776*	0.555-1.085
0.8	0.2	1.5	0.817*	0.591-1.129	0.844*	0.604-1.181

Abbreviations: UC: unmeasured confounder; HR: hazard ratio; OS, overall survival; PFS: progression-free survival.

* Scenarios in which the effects of unmeasured confounder would render the association of treatment group with survival no longer statistically significant.

### Toxicity

Most treatment-related and documented acute toxicities were grade 1 or 2 (Table 5); no treatment-related deaths were observed. Grade 3-4 esophagitis was more common in the PF group than the DP group (16/102 *vs*. 4/102; *P* = 0.008), whereas grade 3-4 thrombopenia and skin toxicities were significantly more common in the DP group than the PF group (3/102 *vs*. 10/102, *P* = 0.045; 7/102 *vs*. 19/102, *P* = 0.020; respectively).

**Table 5:** Acute toxicity

CTC Grade	PF group (*n* = 102)					DP group (*n* = 102)					*P*^a^ (PF *vs*. DP)
	0	1	2	3	4	0	1	2	3	4	
**Anemia**	56	26	13	6	1	55	28	13	5	1	0.774
**Leukocytopenia**	21	20	25	16	20	21	19	14	26	22	0.088
**Thrombopenia**	73	15	10	3	0	65	20	7	8	2	0.045
**Gastrointestinal**	37	33	21	15	0	33	41	9	19	0	0.574
**Skin toxicity**	36	37	22	7	0	29	38	16	19	0	0.020
**Esophagitis**	9	49	28	16	0	13	58	27	4	0	0.008
**Pneumonitis**	70	21	9	2	0	75	18	6	3	0	0.684

a *P*-values were calculated using the chi-squared test to compare patients’ grade 3-4 toxicities between the DP and PF groups.

## DISCUSSION

Limited data comparing the efficacy and toxicity of PF and DP are available in patients with unresectable ESCC. In the current retrospective study, we found that DP may offer a survival advantage over PF and is associated with a lower risk of esophagitis but higher risk of hematological and skin toxicities.

Since the RTOG 85-01 established concurrent chemoradiotherapy as the standard treatment for locally advanced esophageal cancer, PF has been widely used in clinical practice [4, 5, 6]. However, PF offers limited benefit, with a clinical complete response rate of 15-62.2% and 2-year OS rate of 31.5-46% [4, 5, 10–14]. An overview of the literature concerning the efficacy and side-effects of concurrent PF and DP is shown in Supplementary Table S1. Concerns regarding the efficacy and toxicity of PF have led oncologists to investigate alternative regimens, mainly including taxane-based regimens (paclitaxel or docetaxel plus cisplatin). As shown in Supplementary Table S1, taxane-based regimens have also been investigated in several exploratory trials and have shown good efficacy against esophageal cancer, especially ESCC. Several studies have compared taxane-based regimens with the traditional PF regimen, but yielded controversial results. To date, there has only been one published randomized prospective comparison of DP and PF. The study of 90 patients with ESCC undergoing concurrent radiotherapy reported by Zhao et al. [15] found the DP regimen led to a better overall response rate and OS compared to PF (median OS: 43.2 *vs*. 22.3 months, *P* < 0.05). In contrast, another retrospective study reported by Adelstein et al. suggested that a taxane-based regimen led to increased toxicity with no improvement in survival compared to PF [16]. In a multi-center retrospective analysis, Honing et al. assessed the survival outcomes and toxicity of definitive chemoradiotherapy concurrently with carboplatin/paclitaxel or cisplatin/5-fluorouracil. OS and disease-free survival were similar between groups, but the rates of both hematological and non-hematological toxicities were lower in the carboplatin/paclitaxel group [17]. Collectively, the findings reporting the efficacy of taxane-based regimens have been controversial and are complicated by the fact that small case series or retrospective studies make it difficult to reach a definitive conclusion.

In this study, we observed that patients receiving DP had better OS and PFS compared to patients treated with PF in the propensity-matched cohort. To our knowledge, the current study is the largest study to compare survival outcomes and toxicity in patients who received PF or DP for unresectable esophageal cancer. In addition, we used propensity score-matched analysis to reduce bias introduced by the non-random assignment of the two regimens being compared. Further phase III randomized controlled trials are warranted to confirm these results.

Toxicity concerns are an important factor limiting the application of chemoradiotherapy in patients with cancer. The incidence of severe acute toxicity in this cohort was slightly higher than that reported in previous studies of patients who received concurrent DP [7, 9, 18], which may be attributed to the relatively higher radiotherapy dose (50-70 Gy) and different chemotherapy density for this cohort. Additionally, the higher risk of hematologic and skin toxicities in the DP group was manageable and could be mitigated by preventive measures. Therefore, radiotherapy concurrently with DP is a relatively safe treatment option for patients with esophageal cancer.

This study has a number of inherent limitations. The current study is limited by its retrospective nature and the fact it was conducted in a single institution. Although we used propensity score-matched analysis to improve comparability between the two regimens, this analysis cannot replace a prospective randomized trial. In addition, since all patients had ESCC, the results of this analysis may be difficult to extrapolate to patients with esophageal adenocarcinoma. Finally, the DP regimen included either three-weekly or weekly regimens, which may affect the results, although the pharmacokinetics for the weekly and three-weekly regimens are similar [19].

In conclusion, this study indicates that chemoradiotherapy with DP may be a more effective option for ESCC and lead to better survival outcomes than PF. Given the prevalence of unresectable esophageal cancer, large phase III randomized controlled trials are required to definitively confirm these preliminary results.

## MATERIALS AND METHODS

### Patient population

The medical records of 540 consecutive patients diagnosed with pathologically-proven ESCC who received concurrent chemoradiotherapy with either PF or DP at our institution between January 2002 and June 2013 were retrospectively reviewed. The ECOG performance status of patients was determined by doctors and recorded routinely in the medical records when patients were visiting doctors. We excluded 157 patients (29.1%) who received other concurrent chemotherapy regimens or who received other anticancer agents in addition to PF or DP. We also excluded 40 (7.4%) patients who received additional induction chemotherapy and 26 patients (4.8%) diagnosed with distant metastatic disease. The remaining 317 patients were included in this retrospective study. The institutional review board (IRB) of the Cancer Center, Sun Yat-sen University approved this study.

All patients underwent a pretreatment work-up, which included a complete medical history, physical examination, hematological and biochemical profiles, thoracoabdominal computed tomography (CT) scan, barium esophagography, pulmonary function test and endoscopic ultrasonography. Bone scans were performed if clinically indicated. Patients were staged using the 6^th^ edition of the TNM staging system of the American Joint Committee on Cancer. Analysis of comorbidity burden was performed using the Charlson comorbidity index [20].

### Radiotherapy and chemotherapy

A total prescribed dose of 50-70 Gy was delivered to the gross tumor volume (GTV) in 1.8-2.0 Gy per fractions over 5-7 weeks. A prophylactic dose (50-54 Gy) was delivered to the clinical target volume (CTV). The primary gross tumor volume (GTV) and volume of involved lymph nodes (GTV-N) were defined by computerized imaging. The CTV included the GTV with a 3 cm margin in the cephalad and caudal direction and 0.5 cm margin in the lateral and anteroposterior directions. The CTV for the lymph nodes included the GTV-N without an additional margin. The planning target volume (PTV) included the CTV with a 2 cm margin in the superior-inferior directions and a 0.5 cm margin in the lateral direction, as previously described [21].

In the PF group (156 patients), two cycles of 5-fluorouracil and cisplatin were given during radiotherapy at 4-week intervals. Cisplatin (60 mg/m^2^) was administered on day 1 with standard hydration, followed by 5-fluorouracil 300 mg/m^2^/24 h by continuous intravenous infusion on days 1-3 of each cycle [21]. In the DP group, 131 patients received a concurrent three-weekly schedule of docetaxel (60 mg/m^2^ on day 1) plus cisplatin (80 mg/m^2^ on day 1) [8]. Another 30 patients were treated with 25 mg/m^2^ cisplatin and 25 mg/m^2^ docetaxel weekly for 5-7 weeks [22]. In cases of severe hematologic toxicity, dose adjustment was performed in the following chemotherapy cycle.

### Toxicity and follow-up

Chemoradiotherapeutic toxicities were graded according to the National Cancer Institute Common Toxicity Criteria (version 3.0). Patients were followed up *via* physical examination, chest and abdominal CT, digestive endoscopy and barium esophagography at 3-monthly intervals for the first two years and every 6 months thereafter. The date of last follow-up was December, 2014.

### Statistical analysis

Continuous data are presented as mean ± standard deviation (SD). Differences in baseline characteristics between the two groups were compared by standardized differences [23]. Standardized differences ≥ 10% is considered statistical difference. Univariate and multivariate logistic regression were applied to assess the associations between clinicopathologic factors and the chemotherapy regimens. The Pearson χ^2^ test was used to compare treatment-related toxicities between groups.

To reduce bias introduced by the non-random treatment assignment, the nearest-neighbor matching algorithm was used to predict a propensity score and create comparable cohorts of patients to evaluate the effect of the concurrent chemotherapy regimen on prognosis [24]. The following variables were included in the model, regardless of their individual statistical significance: sex, age, era of diagnosis, performance status, weight loss, hemoglobin level, platelet count, serum albumin level, Charlson comorbidity index, histologic grade, tumor location, and TNM stage. Overall survival (OS) was the primary endpoint, and was calculated from the diagnosis of ESCC until death or last follow-up. Progression-free survival (PFS) was the secondary endpoint, and was defined as the duration until locoregional recurrence or distant progression, last follow-up or death. Kaplan-Meier method was applied to estimate survival curves, the differences were compared using the log-rank test. A multivariate analysis using Cox proportional hazards survival regression analyses with stepwise regression (forward selection) for the whole cohort was conducted to evaluate the influence of clinical variables on OS and PFS. To induce balance between groups (DP *vs*. PF) on prognostic factors of outcomes and consolidate the strength of our findings, the hazard ratio (HR) was calculated by three different methods. Firstly, patients were stratified into quintiles based on the propensity scores. The HR was estimated when using a Cox proportional hazards model which stratified on the five propensity score strata. The results were verified with analyses conducted as the following: matching patients in DP and PF groups by propensity score with a caliper of 0.2 in a 1:1 ratio, using an inverse probability treatment weighting (IPTW) method. A marginal structural Cox proportional hazards model was also used to compare OS and PFS between DP and PF groups in the matched cohort. To investigate the potential effects of unmeasured confounders on our results, a sensitivity analysis was conducted [24, 25]. A two-side *P* value < 0.05 was considered statistically significant.

## SUPPLEMENTARY MATERIAL TABLE


