# Supplementary material for: Concurrent cisplatin and 5-fluorouracil versus concurrent cisplatin and docetaxel with radiotherapy for esophageal squamous cell carcinoma: a propensity score-matched analysis
Source: Oncotarget. 2016 May 11;7(28):44686–94. doi: 10.18632/oncotarget.9301 (PMC5190128; doi:10.18632/oncotarget.9301)
Supplement: Supplementary file 1 [file oncotarget-07-44686-s001.pdf]

Concurrent cisplatin and 5-fluorouracil *versus* concurrent cisplatin and docetaxel with radiotherapy for esophageal squamous cell carcinoma: a propensity score-matched analysis

Supplementary Material

Supplementary Table S1:Literature overview of clinical trials concerning PF or DP in definite chemoradiotherapy for esophageal cancer.

Author [Reference]	*No*	Histology	Chemotherapy	Radiotherapy	Response	Survival	Toxicitygr 3-4	
							Hematological	Non-hematological
Cooper [4]	61	SCC:51<br>ADC:10	Cisplatin 75 mg/m2 and 5-Fu 1g/m2	50Gy/25f	By follow-up<br>Complete: 27%,<br>Persistent:26%	5-year OS: 26%		
Minsky [11]	218	SCC:187<br>ADC:31	5-Fu 1g/m2 for4 days and cisplatin 75 mg/m2	A: 64.8Gy<br>B: 50.4Gy		2-year OS:<br>A: 31%;<br>B: 40%	A: 73 (67%);<br>B: 75 (69%)	
Ishida [5]	60	SCC	Cisplatin 70 mg/m2 on days 1, 29 and5-FU 700 mg/m2/day on days 1–4 , 29–32	60Gy/30f	CR: 9 (15.0%)<br>PR: 32 (53.3%)	2-year OS: 31.5%	Leukocyte: 20;<br>Hemoglobin: 4;<br>Platelet:3;<br>Neutrophil: 5	Esophagitis:2; ALT:6;<br>Nausea:3; Infection:1;<br>Dyspnea:2
Bedenne [12]	142	SCC:124<br>ADC:18	5-FU 800 mg/m2/day on Days 1-5 and Cisplatin15 mg/m2 on Days 1-5 for 5 cycles	66Gy/33f or 45 Gy/15f		2-year OS: 36.5%	Leukocytes :26;<br>Febrile neutropenia:3;<br>Hemoglobin:3;<br>Thrombocytes:9	Vomiting:9;<br>Esophagitis:5;<br>Cardiovascular:2;<br>Stomatitis:1
Kato [13]	76	SCC	5-FU 400 mg/m2/day on Days 1-5 and 8-12,	60Gy/30f	CR: 46 (62.2%)	3- 5- year OS:44.7%		Esophagitis :17%;

			and Cisplatin40 mg/m2 on Days 1 and 8 every 5 weeks			and36.8%		Nausea :17%: Hyponatremia :16%,Infection without neutropenia:12%
Nishimura [14]	91	SCC:90<br>ADE:1	A:cisplatin 70 mg/m2 Days 1 and 29and 5-FU 700 mg/m2 Days 1–5 and 29–33<br>B:cisplatin 7 mg/m2 Days1–5, 8–12, 29–33 and 36–40, and 5-FU 250 mg/m2 Days 1–14 and 29–42	60Gy/30f		A: 2- and 5-year OS: 46% and 35%<br>B: 2- and 5-year OS:44% and 22%		
Conroy [15]	133	SCC: 115<br>ADC:18	5-Fu 1000 mg/m2 for 4 days and cisplatin 75 mg/m2	50Gy / 25f	CR: 55 (43%)<br>PR: 28 (22%)	Median OS: 17.5m; median PFS:9.4m	Anaemia: 14; Thrombocytopenia:10 ; Lymphopenia:22; Neutropenia: 37; Febrile neutropenia:9	Dysphagia:31; Asthenia:13;Oesophagitis :11;
Li [9]	59	SCC	docetaxel 60 mg/m2; cisplatin 80 mg/m2 for 2 cycles every three weeks	50–64 Gy / 25–35 f	CR: 71.2%<br>PR: 27.1%	3-year OS: 36.7%	Leukocytopenia:24; Neutropenia: 32; Anemia:1; Thrombocytopenia:5	Esophagitis:5; Pleural effusion: 4; Cough: 2; Liver function:1
Day [25]	24	SCC : 11<br>ADE : 13	docetaxel 15–30 mg/m2 and cisplatin 15–30 mg/m2 per week	50Gy / 25f	CR: 33%<br>PR: 17%	5-year OS: 30.5%<br>5-year PFS: 26.5%	4 (16.6%)	Dysphagia: 9; Nausea: 3<br>Fatigue: 4; Feve: 1<br>Constipation:3
Zhao [17]	90	SCC	DP (n=45): docetaxel 75mg/m2 and cisplatin 75mg/m2 every 28 days;<br>PF (n=45): 5-FU at days 1–4 (250 mg/m2/day), and cisplatin (75 mg/m2) every	50.4Gy / 28f	DP: CR: 40%<br>PR: 33.3%;<br>PF: CR: 28.9%<br>PR: 24.4%	DP:median OS: 43.2m;median PFS: 25.3m;<br>PF:median	DP: 16(35.6%);<br>PF:11 (24.4%)	DP: Esophagitis: 8;Pneumonia:3; Mucositis:1;<br>PF: Esophagitis:

			28 days			OS:22.3m;median PFS:14.0m		9;Pneumonia:2; Mucositis:1;

Abbreviations: DP: docetaxel and cisplatin; PF: cisplatin and fluorouracil; SCC: squamous cell carcinoma; ADE: adenocarcinoma; Fu:fluorouracil; OS: overall survival; PFS: progression-free survival.
